# Education and training as a strategy to improve justification of medical imaging referrals in emergency departments

**DOI:** 10.1002/jmrs.291

**Published:** 2018-09-02

**Authors:** Cláudia Sá Dos Reis, Colleen Bennett, Zhonghua Sun

**Affiliations:** ^1^ Discipline of Medical Radiation Sciences Curtin University Perth Australia

## Abstract

Diagnostic imaging pathways are developed to ensure that medical imaging examinations are appropriately selected and referred by clinicians with the aim of justifying the use of imaging modalities for clinical diagnosis. Failing to comply with the imaging pathways or guidelines results in exposing patients to unnecessary ionising radiation due to malpractice of imaging referrals. This editorial provides a comment on a recent study reporting very high percentage of general x‐ray imaging referrals which did not or partially met the imaging pathways in an emergency department.

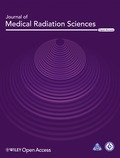

Advances in medical imaging technologies over the past number of decades have resulted in an increase in the sensitivity and specificity of imaging examinations, thereby improving diagnostic accuracy and the opportunity to deliver more accurate and personalised care. Parallel to these improvements, defensive medicine^1^ has also promoted the increased use of medical imaging, which has become a routine diagnostic procedure in clinical practice. Since some of these examinations still involve the use of ionising radiation, it is of paramount importance that radiation protection strategies are implemented to reduce the risks associated with health detriments due to the radiosensitivity of biological tissues. The International Commission on Radiological Protection (ICRP) recommends the adoption of strategies for radiation protection that involves justification, optimisation, training as well as quality control of x‐ray systems.^2^ With justification being the first approach, it is important to ensure that this is well recognised and adhered to by referring medical practitioners (e.g. the patient's physician or surgeon) and medical imaging practitioners (e.g. radiographers and radiologists responsible for conducting the imaging examination). The criteria used in the decision‐making process, which resulted from consensus and research, are not yet fully disseminated but have been introduced in practice in some countries.^3^, ^4^ Furthermore, these criteria only serve as an advisory guidance and are not compulsory, and some healthcare professionals are not familiar with them or with radiation risks.^5^ Consequently, the selection of alternative modalities, which do not use ionising radiation (e.g. ultrasound and magnetic resonance imaging) are sometimes not considered.

In Australia, the Western Australia (WA) Government has developed the Diagnostic Imaging Pathways, which provide evidence‐based medicine to guide health professionals in the judicious use of imaging modalities for diagnosis of various pathologies. This presents the most appropriate diagnostic examinations in a desirable sequence for a wide range of clinical scenarios.^3^ The designed pathways, however, can be adjusted depending on patient presentation, local availability of equipment and expertise as well as the experience of referring clinicians. These imaging pathways ensure that referrals for imaging examinations are appropriate and medically justified. Failing to do so may result in unnecessary imaging examinations and exposure of patients to radiation dose which could be avoided.

In the current issue of the *Journal of Medical Radiation Sciences*, Rawle and Pighills^6^ analysed the number of plain x‐ray imaging examinations performed in an emergency department in a regional Queensland hospital. The purpose of their study was to determine whether referrals for these imaging examinations were medically justified according to the diagnostic imaging pathways developed by the Government of WA. During the audit period of 11 days, authors retrieved 186 referrals for general x‐ray imaging, with imaging of the ankle, knee and shoulder representing the three most frequently performed radiographic procedures (51.6% of all referrals). The corresponding number of examinations that met the imaging pathways was 9, 26 and 46% respectively. Analysis of all referrals indicated that only a quarter (24.7%) of examinations met the diagnostic imaging pathways, while the remaining 75.3% of examinations only partially met or did not meet the imaging pathways. About one‐third of the referrals did not provide clinical information such as the patient's symptoms or signs. Inclusion of these clinical details was found to be significantly associated with the number of referrals meeting the imaging pathway (*P* < 0.001).

Similarly, a review of 140 medical records which did not meet the pathway or were unclear (Fig. 2, Table 2), a high percentage of medical records (65.5%) did not meet the imaging pathway criteria. Further analysis of the data was performed by combining these imaging referrals with medical records, and their results showed improvement with unjustified examinations reduced to 49%. Results of this study indicate that an excessive high percentage of clinically unjustified x‐ray imaging examinations were performed in the emergency department. They concluded that referring clinicians were not providing adequate relevant information and that the radiographers were not complying with justification requirements.

There are several aspects from Rawle and Pighills’ study^6^ that deserve to be discussed. Despite the limitation of a single centre experience, the authors highlighted the significance of excessively high rates of unjustified emergency imaging examinations. With more than 75% of imaging referrals not meeting or only partially meeting the imaging pathways, justification of the use of general x‐ray imaging is not appropriately implemented in clinical practice. Additionally, clinical details related to each patient were not provided in nearly one‐third of examinations. This further emphasises the importance of complying with the guidelines when clinicians select imaging examinations as part of the diagnostic approach.

The WA pathways are intended to help medical practice by improving the efficiency of health service delivery and promoting fair and cost‐effective care; however, its use as a reference brings some limitations. As individual patient circumstances vary, each pathway is neither a rigid set of rules nor a substitute for clinical assessment. It is also acknowledged that diagnostic practice may vary from one healthcare provider to another. Other variables include local availability of equipment and available expertise as previously stated. The 173 individual scenarios available cannot cover all possible patient presentations and for that reason, a divergence from the proposed pathway may be acceptable in certain circumstances.

Another important issue to consider is the lack of research to identify the reasons behind whether it is due to the quality or absence of provided training or is a result of the availability of equipment and expertise in the fields that were explored or the communication between all individuals involved. The information provided to patients and the consent obtained from them to perform an examination may also influence the ultimate approach to diagnosing the patient's condition.^7^ These aspects were not explored and discussed in this study. Malone and others^8^ noted that 20–50% of the imaging performed could not be justified on clinical grounds and the causes were multiple and could vary according to location, specialty requesting, practitioner, quality and frequency of training, availability and quality of referral criteria.

In summary, one strategy that can be considered to reduce the number of unjustified examinations being performed is to develop criteria that are adapted to each context to guide the respective clinical community and to simultaneously introduce high quality education and training sessions across healthcare departments. The aim of these sessions would be to improve knowledge of radiation exposure in medical imaging, associated risks and appropriate examinations available for main pathological conditions based on clinical audits.
